# Measuring women’s childbirth experiences: a systematic review for identification and analysis of validated instruments

**DOI:** 10.1186/s12884-017-1356-y

**Published:** 2017-06-29

**Authors:** Helena Nilvér, Cecily Begley, Marie Berg

**Affiliations:** 1 Institute of Health and Care Sciences, Sahlgrenska Academy, University of Gothenburg, Gothenburg, Sweden; 2 School of Nursing and Midwifery, Trinity College Dublin, Dublin, Ireland; 3 Centre for Person-Centred Care (GPCC), University of Gothenburg, Gothenburg, Sweden

**Keywords:** Systematic review, Validated questionnaires, Measurement instruments, Psychometric properties, Childbirth experiences, Childbirth satisfaction

## Abstract

**Background:**

Women’s childbirth experience can have immediate as well as long-term positive or negative effects on their life, well-being and health. When evaluating and drawing conclusions from research results, women’s experiences of childbirth should be one aspect to consider. Researchers and clinicians need help in finding and selecting the most suitable instrument for their purpose. The aim of this study was therefore to systematically identify and present validated instruments measuring women’s childbirth experience.

**Methods:**

A systematic review was conducted in January 2016 with a comprehensive search in the bibliographic databases PubMed, CINAHL, Scopus, The Cochrane Library and PsycINFO. Included instruments measured women’s childbirth experiences. Papers were assessed independently by two reviewers for inclusion, and quality assessment of included instruments was made by two reviewers independently and in pairs using Terwee et al’s criteria for evaluation of psychometric properties.

**Results:**

In total 5189 citations were screened, of which 5106 were excluded by title and abstract. Eighty-three full-text papers were reviewed, and 37 papers were excluded, resulting in 46 included papers representing 36 instruments. These instruments demonstrated a wide range in purpose and content as well as in the quality of psychometric properties.

**Conclusions:**

This systematic review provides an overview of existing instruments measuring women’s childbirth experiences and can support researchers to identify appropriate instruments to be used, and maybe adapted, in their specific contexts and research purpose.

**Electronic supplementary material:**

The online version of this article (doi:10.1186/s12884-017-1356-y) contains supplementary material, which is available to authorized users.

## Background

Childbirth experiences can have immediate as well as long-term positive or negative effects on life, well-being and health [1]. A positive experience can be remembered as an empowering life event [1–3] connected to personal growth and self-knowledge affecting the transition to motherhood [4]. A negative birth experience increases the risk of negative health outcomes, such as postpartum depression [5] and future fear of giving birth [6], that can lead to a request for caesarean birth in future pregnancies [7, 8], and have an impact on future reproduction [9, 10]. The memory of a birth can vary over time for the woman, with either more positive or negative memories being recalled at a later period after birth compared to directly after [3, 11]. Furthermore childbirth, as experienced by the woman giving birth, can vary considerably from how a caregiver or relative may experience the same event. The person beside the woman may focus on more tangible, observable aspects and underestimate psychological aspects. It is therefore important that women are asked for their experiences [12]. Women have the right to a dignified, respectful, and humane health care during childbirth. Mistreatment of women in childbirth is a violation of women’s fundamental human rights [13]. Such mistreatment can occur both in the interaction between the woman and health care provider as through systematic failures in health facilities and health system levels. Therefor there is need of reliable and validated instruments to highlight women’s experiences and promote respectful and supportive care [14].

Studies on women’s childbirth experiences have been using different surrogate terms and related concepts such as ‘childbirth satisfaction’, ‘satisfaction with care’, ‘experiences of control’ or ‘of support’, ‘experience of relationship with caregivers’ and ‘experience of pain’ [15]. Women’s satisfaction with childbirth is multidimensional and affects the childbirth experience [16]. When evaluating and drawing conclusions from care in labour and birth, women’s experiences of childbirth should be one outcome of considerable importance to measure. This requires the use of reliable and valid instruments adapted to the purpose. As researchers might select and use different terms related to each other when studying women’s childbirth experiences, we have chosen to include instruments that use surrogate terms and related concepts in this review.

For an instrument to receive good levels of reliability and validity, extensive development and testing of psychometric properties is needed [17]. Without valid psychometric properties, conclusions drawn may be false and lead to invalid conclusions on the concept [18].

No review specifically focusing on instruments measuring women’s childbirth experiences has been found, but there are two reviews evaluating instruments measuring ‘maternal childbirth satisfaction’ [19, 20]. Perriman and Davis identified and reviewed 4 instruments measuring maternal satisfaction with continuity of maternity care models in before, during and after labour and birth. The papers describing the instruments primarily compared outcomes rather than describing the development of the tool [19]. Sawyer et al. identified and reviewed 9 multi-item instruments specifically studying maternal satisfaction with care given during labour and birth [20]. In an attempt to give researchers and clinicians an overview, we performed a systematic review to identify and present validated instruments measuring women’s childbirth experience.

## Methods

A systematic review is a rigorous method of research that follows a systematic procedure to enable a summary of all findings from multiple studies on a specific topic. The start point is a rigorous search process for capturing the entire body of scientific studies [21]. As researchers might select and use different terms related to each other when studying women’s childbirth experience [15], we have chosen to use a broad definition and use surrogate terms and related concepts in this review, e.g. childbirth satisfaction, control, support, fear. The Cochrane guideline was used as guidance [21].

### Eligibility criteria

First a review protocol was developed (see Additional file1). Inclusion and exclusion criteria were established in advance and documented in the review protocol. Criteria for inclusion in this review were as follows:

- Papers representing instruments measuring women’s childbirth experience.
- Papers should describe the development or test psychometric properties of an instrument.
- Instruments assessing both pregnancy, childbirth and the postpartum period are included if one or more dimensions are related to women’s childbirth experiences, and this could be assessed as a separate scale.
- Papers reporting original research, published in peer-reviewed journal.
- Reviews were included to enable us to find original papers.
- Papers published in English or French were included as the researchers could understand these languages.

Dissertations, non-original research, or conference papers were excluded.

### Search strategy

The search strategy was designed and developed following consultation with a healthcare librarian. Before the final search all authors commented and agreed on the search string that was adapted for the individual databases (see Additional file 2). The final search took place in January 2016 in the electronic databases of PubMed, Scopus, CINAHL, Cochrane Library and PsycINFO. No restriction in the dates of publishing was made.

**Fig. 1:**
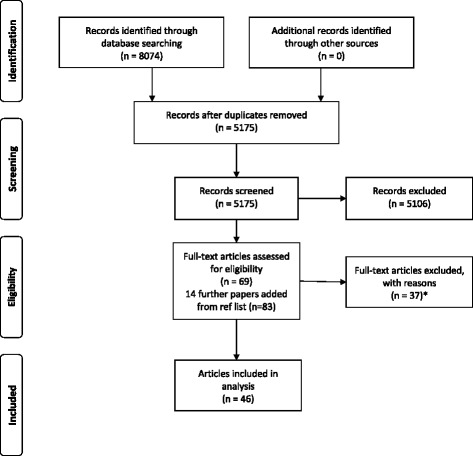
Flow chart of study selection

In total 8074 citations were identified (PubMed *n* = 2785, CINAHL *n* = 1140, PsycINFO *n* = 558, Scopus *n* = 3426 and Cochrane *n* = 165). For the initial screening all the search results were imported into reference management software (EndNote) and duplicates were removed, leaving 5106 titles and abstract to be screened for inclusion. First, papers clearly irrelevant to our topic, such as papers assessing childhood development, contraceptives etc., were removed by one of us (HN). The remaining 809 titles and abstract were assessed independently by two researchers (HN and an assistant, JC). This identified 266 residual papers which were assessed independently by two of the reviewers (HN and MB) to include papers for more in-depth full text assessment. Sixty-nine papers were retrieved in full text and assessed for eligibility criteria by two reviewers independently (HN and CB, or MB and CB, or HN and MB). Any potential conflicts were solved by the third reviewer. Fourteen additional studies were found through search of reference lists of included papers and were assessed in full-text by two independent reviewers for eligibility criteria (HN and MB). Three of these papers were included after assessment in full text. In total 83 papers were thus assessed in full text of which 37 did not fulfil the inclusion criteria and were excluded with reason (see Table 1). The names of each instrument were then searched in PubMed and CINAHL to retrieve further potential papers related to the specific instrument. No further papers on the development or testing of psychometric properties of the identified instruments were found. The flow of selection for studies are shown in Fig. 1.

**Table 1:** Excluded papers with reason

Instrument	Reason for exclusion
Bowers BB: Development of an instrument to measure mothers’ perceptions of professional labor support. Texas Woman’s University; 2001.	Dissertation
Callahan JL, Hynan MT: Identifying mothers at risk for postnatal emotional distress: further evidence for the validity of the perinatal posttraumatic stress disorder questionnaire. J Perinatol 2002; 22(6):448–454.	Focus on postnatal medical complications of infant in relation to mothers health rather than on childbirth experiences
Chen CH, Wang SY: Women’s perceptions of caesarean delivery. Gaoxiong Yi Xue Ke Xue Za Zhi 1992; 8(5):241–246.	In Chinese
Claudia Uribe T, Aixa Contreras M, Luis Villarroel D: Adaptation and validation of the Maternal Welfare Scale in childbirth situations: Second version for integral assistance scenarios. Revista Chilena de Obstetricia y Ginecologia 2014; 79(3): 154–160.	In Spanish
Claudia Uribe T, Aixa Contreras M, Luis Villarroel D, Soledad Hivera M, Paulina Bravo V, Marieta Cornejo A: Maternal wellbeing during childbirth: Development and application of a measurement scale. Revista Chilena de Obstetricia y Ginecologia 2008; 73(1):4–10.	In Spanish
Declercq ER, Sakala C, Corry MP, Applebaum S: Listening to Mothers II: Report of the Second National U.S. Survey of Women’s Childbearing Experiences: Conducted January-February 2006 for Childbirth Connection by Harris Interactive(R) in partnership with Lamaze International. J Perinat Educ 2007; 16(4):9–14.	Not able to distinguish childbirth experience as separate scale from rest of questionnaire.
De Holanda CSM, Alchieri JC, Morais FRR, De Oliveira Maranhão TM: Strategies for development, follow-up, and assessment of care provided to women in the pregnancy-postnatal cycle. Revista Panamericana de Salud Publica/Pan American Journal of Public Health 2015; 37(6):388–394.	In Portuguese
Drummond J, Rickwood D: Childbirth confidence: validating the Childbirth Self-Efficacy Inventory (CBSEI) in an Australian sample. J Adv Nurs 1997; 26(3):613–622	Measures expectancies of labour
Denis A, Séjourné N, Callahan S: Étude de validation française de la version courte du Maternal Self-report Inventory. L’Encéphale: Revue de psychiatrie clinique biologique et thérapeutique 2013; 39(3):183–188.	Not able to separate childbirth experience from the rest of the questionnaire.
Garthus-Niegel S, Storksen HT, Torgersen L, Von Soest T, Eberhard-Gran M: The Wijma Delivery Expectancy/Experience Questionnaire: a factor analytic study. J Psychosom Obstet Gynaecol 2011; 32(3):160–163.	To assess fear of childbirth during pregnancy
Harvey S, Rach D, Stainton MC, Jarrell J, Brant R: Evaluation of satisfaction with midwifery care. Midwifery 2002; 18(4):260–267.	Not specifically on the childbirth experience
Hung CH, Hsu YY, Lee SF: Couples’ satisfaction with health care service during labor and delivery. Kaohsiung J Med Sci 1997; 13(4):255–262.	Assess couples’ experience, not able to distinguish women’s experiences.
Ip WY, Chan D, Chien WT: Chinese version of the Childbirth Self-efficacy Inventory. J Adv Nurs 2005, 51(6):625–633.	Measures expectancies of labour
Ip WY, Chung TK, Tang CS: The Chinese Childbirth Self-Efficacy Inventory: the development of a short form. J Clin Nurs 2008; 17(3):333–340.	Measures expectancies of labour
Janssen PA, Dennis C, Reime B: Development and psychometric testing of the Care in Obstetrics: Measure For Testing Satisfaction (COMFORTS) scale. Research in Nursing & Health 2006, 29(1):51–60 10p.	Not able to distinguish childbirth experience so that it can qualify as a scale of its own
Khalatbari J, Ghasemabadi E, Ghorbanshirodi S: Effect of early Skin-to-skin contact of mother and newborn on mother’s satisfaction. Life Science Journal 2013; 10(SUPPL.3):423–425.	No psychometric analyses
Kishi R, McElmurry B, Vonderheid S, Altfeld S, McFarlin B, Tashiro J: Japanese Translation and Cultural Adaptation of the Listening to Mothers II Questionnaire. J Perinat Educ 2011; 20(1):14–27.	Not able to distinguish childbirth experience from the rest of the questionnaire.
Lee ML, Cho JH: [Development of a scale to measure the self concept of cesarean section mothers]. Kanho Hakhoe Chi 1990; 20(2):131–141.	In Korean
Lowe NK: Maternal confidence for labor: development of the Childbirth Self-Efficacy Inventory. Res Nurs Health 1993; 16(2):141–149.	Measures expectancies of childbirth
Mas-Pons R, Barona-Vilar C, Carregui-Vilar S, Ibanez-Gil N, Margaix-Fontestad L, Escriba-Aguir V: [Women’s satisfaction with the experience of childbirth: validation of the Mackey Childbirth Satisfaction Rating Scale]. Gac Sanit 2012; 26(3):236–242.	In Spanish
Padawer JA, Fagan C, Janoff-Bulman R, Strickland BR, Chorowski M: Women’s psychological adjustment following emergency cesarean versus vaginal delivery. Psychology of Women Quarterly 1988; 12(1):25–34.	Limited testing and description of psychometric properties. The childbirth Perception Questionnaire is further validated by Bertucci et al. (2012) which is included in the review
Perriman N, Davis D: Measuring maternal satisfaction with maternity care: A systematic integrative review: What is the most appropriate, reliable and valid tool that can be used to measure maternal satisfaction with continuity of maternity care? Women Birth 2016.	Review
Redshaw M, Martin C, Rowe R, Hockley C: The Oxford Worries about Labour Scale: women’s experience and measurement characteristics of a measure of maternal concern about labour and birth. Psychol Health Med 2009;14(3): 354–366	Not experiences of childbirth but on worries about childbirth
Rini EV: The Development and Psychometric Analysis of an Instrument to Measure a Woman’s Experience of Childbirth. West Virginia University; 2014.	Dissertation
Ross-Davie MC, Cheyne H, Niven C: Measuring the quality and quantity of professional intrapartum support: testing a computerised systematic observation tool in the clinical setting. BMC Pregnancy Childbirth 2013; 13:163.	Not the woman’s perspective
Rudman A, El-Khouri B, Waldenstrom U: Women’s satisfaction with intrapartum care - a pattern approach. J Adv Nurs 2007, 59(5):474–487.	Compare different dimensions of the childbirth experience to see how they form different patterns of satisfaction
Salmon P, Miller R, Drew NC: Women’s anticipation and experience of childbirth: the independence of fulfillment, unpleasantness and pain. Br J Med Psychol 1990; 63 (Pt 3):255–259.	Compares antenatal anticipations of childbirth to postnatal experiences of childbirth
Sapountzi-Krepia D, Raftopoulos V, Tzavelas G, Psychogiou M, Callister LC, Vehvilainen-Julkunen K: Mothers’ experiences of maternity services: internal consistency and test-retest reliability of the Greek translation of the Kuopio Instrument for Mothers. Midwifery 2009; 25(6):691–700.	Focus on expectations on childbirth not on experiences
Sawyer A, Ayers S, Abbott J, Gyte G, Rabe H, Duley L: Measures of satisfaction with care during labour and birth: a comparative review. BMC Pregnancy Childbirth 2013; 13:108.	Review
Sinclair M, O’Boyle C: The Childbirth Self-Efficacy Inventory: a replication study. J Adv Nurs 1999; 30(6):1416–1423.	Measures expectancies of childbirth
Stahl K: [Revalidation of a questionnaire assessing women’s satisfaction with maternity care in hospital]. Psychother Psychosom Med Psychol 2010; 60(9–10): 358–367.	In German
Stevens NR, Hamilton NA, Wallston KA: Validation of the multidimensional health locus of control scales for labor and delivery. Res Nurs Health 2011; 34(4):282–296	Pregnant women’s expectations
Sweetser L: Satisfaction with childbirth: measurement and causes. Other titles: 1976; 45(4):163–180.	No psychometric analyses
Takegata M, Haruna M, Matsuzaki M, Shiraishi M, Murayama R, Okano T, Severinsson E: Translation and validation of the Japanese version of the Wijma Delivery Expectancy/Experience Questionnaire version A. Nurs Health Sci 2013; 15(3):326–332.	Assesses pregnant women’s expectations
Tanglakmankhong K, Perrin NA, Lowe NK: Childbirth Self-Efficacy Inventory and Childbirth Attitudes Questionnaire: psychometric properties of Thai language versions. J Adv Nurs 2011; 67(1):193–203.	For pregnant women measuring expectations of childbirth
Tokiwa Y, Kunikiyo K: Literature review on self evaluation of childbirth experience. Kitakanto Medical Journal 2006; 56(4):295–302.	In Japanese
Zweig S, Kruse J, LeFevre M: Patient satisfaction with obstetric care. J Fam Pract 1986; 23(2):131–136.	No psychometric analyses

### Quality assessment of included instruments

As the aim of this review was to identify and assess instruments measuring women’s childbirth experiences, the focus was not on the quality of the studies of the included articles but to identify psychometric properties of identified instruments. This was done using criteria specified by Terwee et al. [17] which refer to the following properties; Content validity, Internal consistency, Criterion validity, Construct validity, Reproducibility agreement, Reproducibility reliability, Responsiveness, Floor and ceiling effects, and Interpretability. The properties were evaluated as; + = positive rating, ? = indeterminate rating, − = negative rating, and 0 = no information available. Terwee et al. emphasise the importance of a clear design and method, and that the sample size needs to be greater than 50 subjects in every subgroup of the analysis [17]. In addition to quality assessment of these properties we added another two criteria. The first one considers the need for the instrument and, for a positive rating, a search for existing instruments had to have been done, demonstrating the need to develop and test a new instrument. The second rating item added is related to face validity. For a positive rating, members of the target population should have been asked about the appropriateness of the questionnaire and of each question.

This rating of the measurement properties was performed independently by two review authors (HN and MB, or HN and CB, or MB and CB). When ratings differed between the pairs, it was discussed and, when conflict remained, the third reviewer was included in the discussion to reach consensus. An overview of the results of the quality rating of psychometric properties of included instruments is displayed in Table 2. The last column in the table gives the total figure awarded to each tool, based on a mark of 1 for each ‘+’, and 0.5 for one or more ‘?’ grades. This is only a rough guide to the overall quality of the instrument and must be interpreted with caution. For example, two tools that both received a mark of 6 may be of very different quality, depending on the criteria that were awarded the points.

In conducting this review, our focus and aim was on identifying measures and conducting a broad assessment of their psychometric properties. Given the large number of instruments found, and their very different foci, it was not possible to make clear recommendations as to one particular instrument that would suit all purposes. Instead, some general suggestions are made as to the instruments that appear to be emerging as the top ranking tools in terms of the quality measurement performed, and the overall mark given.

### Data extraction and analysis

The following data were extracted for each instrument: Name of instrument/acronym, authors (year), country of origin, aim/motive of instrument, number of items, dimensions/subscales, response scale, timeframe to answer the questionnaire, whether or not the questionnaire was available and a short narrative summary of included instruments. The data extraction was made by the first author (HN) and then checked by the other authors for accuracy.

One of the individual papers was conducted by one of the authors (MB). To avoid conflict of interest this paper was assessed for eligibility criteria, and quality assessment was made, by the two other authors (HN and CB).

## Results

Forty-six articles presenting 36 instruments [22–59] measuring women’s childbirth experiences were included for quality assessment. Different surrogate terms and related concepts used in identified instruments were described by authors as: childbirth experience (27.8%), satisfaction with care/birth/childbirth (36.1%), perception of birth/care (13.9%), control (11.1%), support (8.3%), fear of childbirth (5.6%), childbirth trauma (2.8%), birth memories (2.8%) and childbirth schema (2.8%). In five of the identified instruments we found cultural validation/translation of the instrument had been done. Most of the instruments were developed and tested in the United States (6) and in the United Kingdom (6). Further countries represented were: Canada (4), the Netherlands (4), Turkey (3), Sweden (3), Jordan (3), France (2), Italy (2), Australia (1), Senegal (1), and Norway (1). Number of items in the instruments varied from three to 145. Nine of the instruments were uni-dimensional, and 27 consisted of several dimensions/subscales. Quality ratings of psychometric properties are presented in Table 2. Descriptive data of included instruments are presented in Table 3, and characteristics in Table 4. Instruments are reported in alphabetical order by first author.

**Table 2:** Quality rating of psychometric properties with Terwee et al.’s criteria

Instrument	Psycometric properties											Total score
	Need for the instrument	Face validity	Content validity	Internal consist-ency	Criterion validity	Construct validity	Reprod-ucibility (Agree-ment)	Reproc-ucibility (Reliabi-lity)	Respon-sivness	Floor & ceiling effects	Inter-pretata-bility	
The Childbirth Trauma Index [22]	+	0	+	-	0	?	0	0	0	0	0	2.5
The Childbirth Experience Perception Questionnaire [26]	+	+	+	-	+	0	0	?	0	0	0	4.5
The Childbirth Experience Questionnaire [34]	+	+	+	+	0	+	0	+	0	+	0	7
The Survey of Bangladeshi women’s experiences of maternity services [41]	+	+	+	+	0	+	+	0	0	0	0	6
The Birth Companion Support Questionnaire [42]	+	+	+	+	0	+	+	0	0	0	0	6
The Perception of Birth Scale [23, 24]	+	0	+	+	0	0	0	0	0	0	0	3
The Birth Memories and Recall Questionnaire [30]	+	+	+	+	0	0	0	+	0	0	0	5
The Support and Control in Birth questionnaire [25]	+	+	+	+	0	0	0	0	0	0	0	4
A self-administered questionnaire to assess women’s satisfaction with maternity care [43]	+	0	+	+	0	0	+	+	0	0	+	6
The Scale for Measuring Maternal Satisfaction- normal birth [44]	+	+	+	+	+	+	0	0	0	0	0	6
The Scale for Measuring Maternal Satisfaction -caesarean birth [44]	+	+	+	+	+	+	0	0	0	0	0	6
The Labor and Delivery Index [45]	+	+	+	0	0	+	+	+	0	0	0	6
The Labour Agentry Scale [46]	+	0	+	+	0	0	+	+	+	0	0	6
The Birth Satisfaction Scale-Revised [27–29]	+	+	+	+	0	?	0	0	0	0	0	4.5
The Early Labour Experience Questionnaire [47]	?	0	+	+	+	+	0	+	0	0	+	6.5
The Labor and Delivery Satisfaction Index [31]	+	+	+	-	0	+	+	-	0	0	0	5
Women’s delivery experience measures [32]	+	?	+	+	0	+	0	0	0	0	?	5
The maternal satisfaction scale for caesarean section [35]	+	+	+	+	0	+	0	+	0	0	+	7
The Satisfaction with childbirth experience questionnaire [48]	+	+	+	+	0	+	0	0	0	0	+	6
Women’s Perception of Control during Childbirth [48]	+	+	+	+	0	+	0	0	0	0	+	6
The Childbirth Schema Scale [33]	0	0	+	+	0	+	+	0	0	0	+	5
Satisfaction with obstetrical care [49]	+	+	?	+	0	+	0	0	0	+	+	6.5
The Preterm Birth Experience and Satisfaction Scale [50]	+	+	+	+	0	+	0	?	?	0	+	6.5
The Responsivness in Perinatal and Obstetric Health Care Questionnaire [36, 37]	+	+	+	+	0	+	0	0	+	+	+	8
Women’s Satisfaction With Hospital-Based Intrapartum Care Scale [51]	+	+	+	+	0	+	0	0	0	0	+	6
Patient Perception Score [52]	+	+	+	+	+	+	0	0	0	0	+	7
Pregnancy and maternity care patients experiences questionnaire [38]	+	+	+	+	0	+	+	+	+	0	+	9
Women’s view of brith labour satisfaction questionnaire [53]	+	+	+	+	?	+	0	0	0	0	+	6.5
The Perceived Control in Childbirth Scale [54]	+	0	+	+	+	+	0	0	0	0	+	6
The Satisfaction with Childbirth Scale [54]	+	0	+	+	+	+	0	0	0	0	+	6
The Pregnancy and Childbirth Questionnaire [55]	+	+	+	+	?	?	0	+	0	0	+	6.5
The Childbirth Perception Scale [39]	+	+	+	+	0	+	0	+	0	0	+	7
The Scale of Women’s Perception for Supportive Care Given During Labor [56]	+	+	+	+	0	+	0	0	0	0	+	6
The Delivery Fear Scale [57]	+	+	+	+	0	+	?	0	0	0	+	6.5
The Wijma Delivery Expectancy/Experience Questionnaire [40]	+	+	+	+	+	+	0	+	+	0	+	9
The Parental Satisfaction and Quality Indicators of Perinatal Care Instrument [58, 59]	+	+	+	+	0	+	0	0	0	0	+	6

Rating: + = positive, ? = intermediate, − = negative, 0 = no information available, N/A not assessable

**Table 3:** Descriptive data of the included instruments

Name of Instrument/Acronym	Authors (year)	Country	Aim/motive of instrument	Comments
The Childbirth Trauma Index for adolescents/CTI [22]	Anderson (2011)	USA	To determine specific indicators perceived by adolescents as influencing birth trauma.	Developed to aid nurses to assess and direct care to reduce the possibility of a trauma stress response or post-traumatic stress disorder among adolescents postpartum [22]. Further development, adaptation and evaluation of the psychometric properties of this tool would be valuable.
The Childbirth Experience Perception Scale/CEPS [26]	Bertucci et al. (2012)	Italy	To assess women’s perception of their childbirth experience.	A further development of ‘The childbirth perception questionnaire’ [73]. The original questionnaire was excluded from our review as the original paper does not present testing of psychometric properties. Bertucci et al. [26] are aware of this, but they consider the strengths of the questionnaire outweigh the limitations as it takes a broad view of various aspects into consideration when evaluating the childbirth perceptions. The psychometric properties need to to be further evaluated. The validity of the Childbirth experience perception scale was challenged in a letter to Midwifery journal, and the authors replied defending their position [83, 84].
The Childbirth experience questionnaire/CEQ [34]	Dencker et al. (2010).	Sweden	To assess different aspects of first-time women’s perception of their childbirth experience.	Developed to assess different aspects of mothers’ childbirth experiences in order to explore them comprehensively. Suggested \to be used to identify women with negative childbirth experiences and for evaluating quality of care. The development of the instrument is clearly described and primary results of several psychometric properties are presented [34]. The instrument has been validated in the UK [77] and used in research [85].
The survey of Bangladeshi women’s experiences of maternity services/SBWEMS [41]	Duff et al. (2001)	UK	To evaluate satisfaction with maternity care in Sylheti-speaking Bangladeshi women.	This cross-cultural instrument was made by cultural adaptation and translation of an existing measure. This paper can be used as a model and inspiration when developing instruments for use in minority ethnic communities [41].
The Birth Companion Support Questionnaire/BCSQ [42]	Dunne (2014)	Australia	To measure women’s perceptions of social support provided during labour by at least one lay birth companion.	Presents a first rigorous study of this instrument developed to be used in midwifery research [42].
The Perception of Birth Scale/POBS [23, 24]	Fawcett & Knauth (1996) Marut & Mercer (1979)	USA	To measure women’s perceptions of their childbirth experiences.	This questionnaire was originally developed and adapted to measure the perception of women who had vaginal or unplanned caesarean births in 1975 [86] and further adapted by Marut and Marcer [24] in 1979. Attempts have been made to adapt and test psychometric properties [87, 88] before Fawcett and Knauth [23] in 1995 adapted the scale further and made an exploratory factor analysis. The scale needs further tests of its psychometric properties.
The Birth Memories and Recall Questionnaire/BirthMARQ [30]	Foley et al. (2014)	UK	To examine the relationship between memories of birth and postnatal mood and psychopathology.	Developed to measure characteristics of memories of childbirth and to examine the relationship between memories for birth and mental health including emotional and traumatic memories. With further testing of reliability and validity this questionnaire could become a useful tool both in research as well as in clinical practice [30].
The Support and Control in Birth Questionnaire/SCIB [25]	Ford et al. (2009)	UK	To measure support and control in birth.	Focuses on different dimensions of control during childbirth. With further testing of tis psychometric properties it can provide a valid and reliable measure to examine the relationships among support, control, and birth outcomes [25]. It has been culturally validated and translated into Turkish [78].
Women’s satisfaction with maternity care/WSMC [43]	Gerbaud et al. (2003)	France	To measure women’s satisfaction concerning maternity care.	This questionnaire is in French and measure women’s satisfaction with care during pregnancy, hospitalisation for birth, and homecoming. It is tested and developed to be used clinically and evaluated care [43].
The Scale for Measuring Maternal Satisfaction-normal birth/SMMS-normal birth [44]	Gungor & Beji (2012)	Turkey	To measure maternal satisfaction with birth in order to evaluate women’s experiences in labour and the early postpartum period before hospital discharge.	This is a scale developed in two versions, one for normal birth and one for caesarean birth. The scales are constructed to evaluate both the experience of care and the emotional experience of childbirth as a measure of satisfaction. The evaluation of initial psychometric properties are good and with further testing these scales can become a useful tool [44].
The Scale for Measuring Maternal Satisfaction- Caesarean birth/SMMS-caesarean birth [44]	Gungor & Beji (2012)	Turkey	To measure maternal satisfaction with birth in order to evaluate women’s experiences in labour and the early postpartum period before hospital discharge.	See above.
The Labor and Delivery Index/LADY-X [45]	Gärtner et al. (2015)	The Netherlands	A utility measure for economic evaluations in perinatal studies.	Developed to measure cost effectiveness of perinatal care interventions for use in research and is able to discriminate between groups [45]. The only instrument identified that measures economic evaluations in perinatal studies.
The Labour Agentry Scale/LAS [46]	Hodnett & Simmons-Tropea (1987)	Canada	An instrument measuring expectancies and experiences of personal control during childbirth.	Since this scale was developed in 1987 [46] it has been used in studies from a broad range of countries as well as in different types of studies [89–96]. Although widely used, further studies of the psychometric properties are recommended to ensure its validity and reliability.
The Birth Satisfaction Scale - Revised/BSS-R [27–29]	Hollins Martin & Fleming (2011) Hollins Martin et al. (2012) Hollins Martin & Martin (2014)	UK	To measure postnatal women’s birth satisfaction.	The birth satisfaction scale – revised [28] is a further development of the Birth satisfaction scale [27, 29, 97]. The revised version of the scale is a more robust version. They have been used in research [97–99] and further cultural translation and validation has been made in Greece and the US [79, 80, 100].
The Early Labour Experience Questionnaire/ELEQ [47]	Janssen & Desmarais (2013)	USA	To measure women’s experiences with their early labour care.	Developed to measure women’s experience and evaluate care given in the latent and early phase of labour [47, 101]. Additional testing of psychometric properties would strengthen the questionnaire further.
The Labor and Delivery Satisfaction Index/LADSI [31]	Lomas et al. (1987)	Canada	To assess the caring aspects of childbirth care.	Developed for use in clinical trials [31] and has been used in several studies evaluating care given [102–104]. It was developed and evaluated in 1987. Therefore it would be appropriate to perform further testing and updating of its psychometric properties.
Women’s delivery experience measures/MFRM [32]	Mannarini et al. (2013)	Italy	To assess birth experiences after both spontaneous and medically assisted conception.	The statistical analysis was made by using the Rash model with the purpose of defining and validating a latent dimension for birth perception [32].
The maternal satisfaction scale for caesarean section/MSS-caesarean section [35]	Morgan et al. (1999)	Canada	To measure maternal satisfaction in women undergoing elective or non-emergent caesarean section under regional anaesthesia.	Developed by anaesthesiologists and two of the dimensions are measuring satisfaction with anaesthetics and side-effects. It has been properly tested for validity and reliability [35].
The Satisfaction with childbirth experience questionnaire/SWCBE [48]	Oweis (2009)	Jordan	No aim/purpose of the instrument documented.	Oweis [48] developed two scales in the same study to assess women’s childbirth experiences including expectations, satisfaction and self-control. These two scales need further evaluation of their psychometric properties.
Women’s Perception of Control during Childbirth/PCCB [48]	Oweis (2009)	Jordan	No aim/purpose of the instrument documented.	See above.
The Childbirth Schema Scale/CSS [33]	Peirce (1994)	US	To obtain an understanding of schema formation and revision with the known stressor of childbirth.	Developed to gain understanding of the underlying structure of known stressors of childbirth, by comparing the schemas before and after birth [33]. Further development and adaptation of the instrument would strengthen the psychometric properties.
Satisfaction with obstetrical care/SSO [49]	Ramanah (2014)	France Canada Senegal	To measure satisfaction in obstetrical care during labor, delivery and two hours postpartum relevant to the French-speaking context.	This instrument is tested in a French speaking context in Senegal, France and Canada [49]. Further development and evaluation of this instrument would strengthen the validity.
The Preterm Birth Experience and Satisfaction Scale/P-BESS [50]	Sawyer (2014)	UK	To assess parents (women and their partners) experiences and satisfaction with care during very preterm birth (<32 gestational weeks).	Further testing of psychometric properties in larger sample groups would be recommended as well as assessment of when the most suitable time after birth to administer the questionnaire would be [50].
The Responsivness in Perinatal and Obstetric Health Care Questionnaire/ReproQ [36, 37]	Scheerhagen et al. (2015) van der Kooy et al. (2014).	The Netherlands	To evaluating maternal experiences of perinatal care services, using the eight-domain WHO concept.	This questionnaire is based on the eight-domain World Health Organization’s Responsiveness model. The questionnaire has an antepartum version assessing the experience during pregnancy and a postpartum version assessing women’s experiences during childbirth and postpartum care. It has been properly tested for a broad variety of psychometric properties [36, 37, 105]. It has been used to evaluate and compare care [106].
Women’s Satisfaction With Hospital-Based Intrapartum Care Scale [51]	Shaban (2014)	Jordan	To measure women’s satisfaction with intrapartum care in Jordan, especially to examine how low-risk, healthy laboring women experienced are during labor and birth.	Developed to provide information on women’s experiences with the aim of helping caregivers change practices. Further studies evaluating the psychometric properties would be the next step [51].
Patient Perception Score/PPS [52]	Siassakos et al. (2009)	UK	A simple tool to measure maternal satisfaction of operative abdominal and vaginal birth.	This is a short tool adapted from a Patient perception score used in simulation training of obstetric emergency situations and is easy to complete [107]. It aims to capture patient’s perception of operative birth with a focus on perceived communication, respect and safety. This is an easy tool that is suggested by the authors to be used on a regular basis in clinical settings to focus on women’s perceptions and improve care [52].
Pregnancy- and maternity-care patients’ experiences questionnaire./PreMaPEQ [38]	Sjetne (2015)	Norway	To measure women’s experiences of pregnancy and maternity care in Norway and other sites having similar health system.	Developed to collect women’s experiences of the maternity health care system in Norway. It has been well tested for a broad variety of psychometric properties and is an acceptable instrument for collecting women’s experiences of maternity care [38].
Women’s View of Birth Labour Satisfaction Questionnaire/WOMBLSQ [53]	Smith (2001)	UK	To measure maternal satisfaction with care quality of different models of labour care in the UK.	This questionnaire can be used to compare models or systems of labour and care during birth, giving an overall picture of care received. It would strengthen the reliability and validity if the instrument was further evaluated and adapted [53]. It has been culturally translated and adapted in several countries [108, 109] and used in studies [110].
The perceived Control in Childbirth Scale/PCCh [54]	Stevens (2012)	USA	To assess patient perceptions of control of the childbirth environment.	Development of two separate scales in the same paper. A goal of the study was to clarify the theoretical distinctions among similar constructs [54].
The Satisfaction with Childbirth Scale/SWCh [54]	Stevens (2012)	USA	To assess global satisfaction with the childbirth experience.	See above.
The Pregnancy and Childbirth Questionnaire/PCQ [55]	Truijens (2014a)	The Netherlands	To assess quality of care during pregnancy and delivery as perceived by women who recently gave birth.	Two scales, one referring to pregnancy and one referring to birth. Further research and evaluation of the psychometric properties would strengthen the validity and reliability [55]. It has been used in studies [111, 112].
The Childbirth Perception Scale/CPS [39]	Truijens (2014b)	The Netherlands	To assesses the perception of delivery and the first postpartum week.	Developed to compare women’s perception of home and hospital birth [39]. Psychometric properties have been adequate tested but further testing would strengthen validity and reliability.
The Scale of Women’s Perception for Supportive Care Given During Labor [56]	Uludag & Mete (2015).	Turkey	To determine women’s perception of supportive care given during labor.	Developed to see how women perceive care received from nurses to evaluate quality of care [56]. Further evaluation and adaptation of the psychometric properties would strengthen validity and reliability.
Delivery Fear Scale/DFS [57]	Wijma et al. (2002)	Sweden	To measure fear during the process of labor.	This is the only scale that we have identified that has been tested and evaluated for psychometric properties that are meant to be used during labour [80]. The scale has been used in research [113, 114].
The Wijma Delivery Expectancy/Experience Questionnaire/W-DEQ [40]	Wijma et al. (1998)	Sweden	To measure fear of childbirth during pregnancy and after childbirth.	Consists of two versions; one to be used during pregnancy (version A) and one to be used after childbirth (version B) [40]. It has been used extensively [60–66] and cultural validation and translations have been made in several countries [67–69]. It is commonly used for measuring fear of childbirth, and it is properly developed with good psychometric properties.
The Parental Satisfaction and Quality Indicators of Perinatal Care Instrument/PPC [58, 59]	Wool, C. (2015a). Wool, C. (2015b).	US	To measure parental satisfaction and quality indicators in parents electing to continue a pregnancy after learning of a life-limiting fetal diagnosis.	This is the only instrument we identified concerning this subject [58, 59]. Further evaluation of the psychometric properties would strengthen the validity and reliability.

**Table 4:** Characteristics of included instruments

Name of Instrument/Acronym	Items	Dimensions/subscales	Response	Timeframe to answer the questionnaire	Quest-ionnaire available
The Childbirth Trauma Index for Adolescents/CTI [22]	14-items	No	4- point Likert scale and rating of birth experience between 0 and 10	1–3 days postpartum	No
The Childbirth Experience Perception Scale/CEPS [26]	24-items	3 subscales; Labour and Delivery Perception, Control Perception, and Change Perception.	6-point Likert scale	24–48 h postpartum	No
The Childbirth experience questionnaire/CEQ [34]	22-items	4 dimensions; Own capacity, Professional support, Perceived safety, and Participant	4- point Likert scale and VAS	1 month postpartum	Yes
The survey of Bangladeshi women’s experiences of maternity services/SBWEMS [41]	72- items	3 subscales; Ante- (33 items), Peri- (15 items), Post-natal (24 items)	Yes/No, Likert scales and Multiple choice options	2 month postpartum	Yes
the Birth Companion Support Questionnaire/BCSQ [42]	17-items	2 subscales; Emotional support, tangible support	4-point Likert scale	On postnatal ward before discharge	No
The Perception of Birth Scale/POBS [23, 24]	25-items	5 subscales; Labor Experience, Delivery Experience, Delivery Outcome, Partner Participation, and Awareness	5-point Likert scale	1–2 days after birth	No
The Birth Memories and Recall Questionnaire/BirthMARQ [30]	23-itmes	6 dimensions; Emotional memory, centrality of memory to identity, Coherence, Reliving, Involuntary recall, and Sensory memory	7-point Likert scale	Within 1 year after giving birth	Yes
The Support and Control in Birth Questionnaire/SCIB [25]	33-items	3 subscales; Internal control (10 items), external control (11 items), Support (12 items)	5-point Likert scale	On average, 1 year after birth	Yes
Women’s satisfaction with maternity care/WSMC [43]	44-items	11 dimensions	Likert scales and Multiple choice options	2 month postpartum	Yes
The Scale for Measuring Maternal Satisfaction-normal birth/SMMS-normal birth [44]	43-items	10 subscales; perception of health professionals, nursing/midwifery care in labour, comforting, information and involvement in decision making, meeting baby, postpartum care, hospital room, hospital facilities, respect for privacy, meeting expectations	5-point Likert scale	Within 24 h	No
The Scale for Measuring Maternal Satisfaction- Caesarean birth/SMMS-caesarean birth [44]	42-items	10 subscales; perception of health professionals, preparation for caesarean, comforting, information and involvement in decision making, meeting baby, postpartum care, hospital room, hospital facilities, respect for privacy, meeting expectations	5-point Likert scale	Within 72 h	No
The Labor and Delivery Index/LADY-X [45]	7-items	7 domains; Availability, Information, Needs, Emotional support, Worries, Safety, time to first contact with baby	3-point Likert scale	6–8 weeks postpartum	Yes
The Labour Agentry Scale/LAS [46]	29-items	No	7-point Likert scale	Within 72 h postpartum	No
The Birth Satisfaction Scale - Revised/BSS-R [27–29]	10-items	3 subscales: Quality of care provision (4 items), women’s personal attributes (2 items), stress experienced during labour (4 items).	5-point Likert scale	Within 10 days postpartum	Yes
The Early Labour Experience Questionnaire/ELEQ [47]	22-items	3 subscales: Emotional Well-Being (8), Emotional Distress (8), Perception of Nursing Care (6)	5-point Likert scale	During postpartum stay at hospital	Yes
The Labor and Delivery Satisfaction Index/LADSI [31]	38-items	No	6-point Likert scale	2 days postpartum and 4.6 weeks postpartum	Yes
Women’s delivery experience measures/MFRM [32]	31-items	7 dimensions	4-point Likert scale	24–48 h postpartum	No
The maternal satisfaction scale for caesarean section/MSS-caesarean section [35]	22-items	3 subscales: Anaesthetic (6 items), Side-effects (6 items), Atmosphere (10 items)	7-point Likert scale	Not reported	Yes
The Satisfaction with childbirth experience questionnaire/SWCBE [48]	32-items	No	5-point Likert scale	Not reported	Yes
Women’s Perception of Control during Childbirth/PCCB [48]	23-items	No	5-point Likert scale	Not reported	Yes
The Childbirth Schema Scale/CSS [33]	16-item pairs	3 factors: Emotions of outcome (6 items), Sensation of the work of childbirth (4 items), Time (3 items), Preparation for control (3 items)	7-point Likert scale	1 month before and 2 weeks after birth	No
Satisfaction with obstetrical care/SSO [49]	49- items	5 dimensions: Nurse (14), doctor (14), anaesthetist (5), environment (9), global satisfaction (7)	10-point Likert scale	48 h postpartum	Yes
The Preterm Birth Experience and Satisfaction Scale/P-BESS [50]	17-items	3 dimensions: Staff professionalism and empathy, Information and explanations, Confidence in staff	5-point Likert scale	Up to 12 months postpartum	No
The Responsivness in Perinatal and Obstetric Health Care Questionnaire/ReproQ [36]	40-items	8 domains: Dignity, Autonomy, Confidentiality, Communication, Prompt attention, Social consideration, Basic amenities, Choice and continuity.	Not reported	6 weeks postpartum	Yes
Women’s Satisfaction With Hospital-Based Intrapartum Care Scale [51]	14-items	3 dimensions: Interpersonal care (5 items), Information and decision making (4 items), Physical birth environment (5 items)	Not reported	2 months postpartum	No
Patient Perception Score/PPS [52]	3-items	3 items; communication, respect and safety	5-point Likert scale	Within 24 h of birth	yes
Pregnancy- and maternity-care patients’ experiences questionnaire./PreMaPEQ [38]	145-items in total	4 parts in the questionnaire. One of these is Birth and have 3 subscales: Personal relationships in the delivery ward, Resources and organisation in the delivery ward, Attention to partner in the delivery ward.	5 point Likert scal for single items and index scores were transformed linearly to a scale of 0–100.	From 17 weeks after birth	Yes
Women’s View of Birth Labour Satisfaction Questionnaire/WOMBLSQ [53]	Not reported	10 dimensions in addition to general satisfaction	Not reported	Within 10 days of birth	No
The perceived Control in Childbirth Scale/PCCh [54]	12- items	No	6-point Likert scale	Prior to discharge	Yes
The Satisfaction with Childbirth Scale/SWCh [54]	7-items	No	7-point Likert scale	Prior to discharge	Yes
The Pregnancy and Childbirth Questionnaire/PCQ [55]	25-items	Two scales: 18-items referring to pregnancy, 7-items referring to personal treatment during delivery.	5-point Likert scale	Within 6 weeks of birth	No
The Childbirth Perception Scale/CPS [39]	12-items	2 dimensions; Perception of delivery (6-items), perception of first postpartum week (6-items)	4-point Likert scale	7 days postpartum	Yes
The Scale of Women’s Perception for Supportive Care Given During Labor [56]	33-items	3 subdimensions: Comfortable Behaviours (15-items), Education (8-items), Disturbing Behaviours (10 items)	4-point Likert scale	Not reported	No
The Delivery Fear Scale/DFS [57]	10-items	No	10-point scale	During any moment of labor and delivery	Yes
The Wijma Delivery Expectancy/Experience Questionnaire/W-DEQ [40]	29-items	No	6-point Likert scale	Within 2 h of birth and 5 weeks after birth	Yes
The Parental Satisfaction and Quality Indicators of Perinatal Care Instrument [58, 59]	Intra-partum scale: 37 items Post-natal scale include an addit-ional 7 items	3 scales: The Prenatal, The Intrapartum, The Postnatal Scale 8 domains: Structure and processes of care, physical aspects of care, psychological and psychiatric aspects of care, social aspects of care, spiritual, religious, and existential aspects of care, cultural aspects of care, care of the imminently dying patient, and ethical and legal aspects of care.	7-point Likert scale	Not reported	No

A few of the tools gained a low quality rating, which would indicate the need for further development and evaluation of their psychometric properties. These included: *The Childbirth Trauma Index for adolescents* [22] (overall quality mark of 2); *The Perception of Birth Scale* [23, 24] (overall quality marks of 3); *Support and Control in Birth* [25] (overall quality marks of 4); *The Childbirth Experience Perception Questionnaire* [26] and *The Birth satisfaction scale* and *the Birth satisfaction scale - revised* [27–29] (overall quality marks of 4.5); *The Birth Memories and Recall Questionnaire* [30], *The labour and delivery satisfaction index* [31] (an instrument developed and evaluated in 1987, and in need of further testing and updating of its psychometric properties), the *Women’s delivery experience measures* [32], and the *Childbirth schema scale* [33] (overall quality marks of 5).

In general, we would suggests that tools with marks of 2 to 4.5 are not suitable for use without further testing, especially if there is another existing tool that will serve the same purpose. Tools with a mark of 5 may be suitable if they are the only instrument developed in that topic area, but not otherwise, and further testing before use is recommended.

The majority of tools (20 out of 36, 56%) had marks of 6 or 6.5, which probably indicates a suitable tool, unless there is a higher quality one in the same area. We suggest that the seven instruments with marks of 7 to 9 (Table 2) can be considered valid and reliable although, of course, further testing is always welcome and could improve them further. These included: *The Childbirth Experience Questionnaire * [34], *The maternal satisfaction scale for caesarean section * [35], *The Responsiveness in Perinatal and Obstetric Health Care Questionnaire* [36, 37], *Pregnancy and maternity care patients experiences questionnaire* [38] and *The Childbirth Perception Scale *[39]. The tool with the highest quality rating, of 9, was the *Wijma Delivery Expectancy/experience Questionnaire* [40], an instrument measuring fear specific to labour and childbirth with one version used during pregnancy (version A) and one used after childbirth (version B). The Wijma Delivery Expectancy/experience questionnaire has been used extensively [60–66] and cultural validation and translations have been made in several countries [67–69]. As this scale is commonly used for measuring fear of childbirth, and it is properly developed with good psychometric properties, we recommend this scale for measuring women’s experience of fear in childbirth, when a detailed survey is necessary. However, a number of different cut-off points are used to define severe fear of childbirth, resulting in different prevalence rates, and these should be standardised.

## Discussion

The purpose of this systematic review was to identify and analyse instruments that measure women’s childbirth experiences, and 46 papers representing 36 instruments were identified and included. By including surrogate terms and related concepts to the childbirth experiences, a broader and more holistic overview of existing instruments was achieved. Identified instruments demonstrated a wide range in purpose and content as well as in the quality of psychometric properties.

When choosing between different instruments, one needs to consider all ratings together as well as taking into account those measurement properties that are most important for a specific application, setting and population, e.g. practical aspects such as burden for women, and cost and quality aspects regarding the validity and reliability of the instrument [70]. If the researcher chooses an inappropriate or poor quality measurement instrument, this may lead to bias in the conclusion, resulting in wasted resources and unethical procedures for the women that participated [71]. Rudman [72] concluded that a multi-item instrument including different dimensions of care instead of a single global measure, gave a more diverse and richer picture of women’s childbirth experiences but also led to a more negative picture [72]. To choose the right instrument for clinicians and researchers for their specific context is a complex process. In our result we present an overview in Tables 1 and 2 of descriptive data and characteristics of instruments as well as a narrative summary of the individual instruments, which can aid in this process.

Terwee et al. [17] consider the content validity to be the single most important psychometric property of the questionnaire, and state that only if the content validity is adequate can the questionnaire be considered, and the remaining measurement properties become useful. All instruments in our review did get a positive rating of content validity. But a more thorough investigation would still be advisable to see which instruments have the strongest content validity to aid in choosing an appropriate instrument. Many of the instruments that we identified would need further testing of their psychometric properties to determine which would be best. This is consistent with the finding of Sawyer et al. [20], who evaluated nine questionnaires about women’s satisfaction during labour and birth, concluding that none of the questionnaires had optimal testing of validity and reliability. Most of the instruments in our review did report on several tests of psychometric properties, but further evaluation of validity and reliability was needed.

Among the excluded papers (Table 1) there are several questionnaires developed that were not included in this review as they did not report on psychometric properties [73] or the focus was on a study rather than development of the instrument [72, 74]. Before using a specific instrument, we suggest that a thorough investigation of the development and testing of the instrument should be done to ensure good psychometric properties. In the US Food and Drug Administration’s guidelines on developing new patient-reported outcome measures, they suggest that a new instrument can be developed by modifying an existing one [18]. As we found a large number of questionnaires and instruments, we agree with this suggestion. When conducting studies of psychometric properties of an instrument, we recommend applying standards such as the COSMIN checklist [75, 76] and Terwee et al.’s criteria [17] in order to enhance the quality of the results and to facilitate the researcher to compare and find an instrument with good psychometric properties.

Several of the papers included in our review consisted of development and validation of existing questionnaires [23, 26, 41]. As well, several of the questionnaires have been culturally translated and validated in other languages and cultures [67–69, 77–80].

### Methodological considerations

The attempt with this review was to identify all studies and instruments that meet the eligibility criteria, but it is possible that we have missed relevant articles, written in other languages than English and French, or indexed in other databases than those chosen. A limitation of this search was that we did not use Terwee et al’s PubMed search filter [81] which may have generated more papers. We suggest that this review can be used as a tool for identification of existing instruments, while acknowledging that each researcher will have to assess their chosen tool themselves in the light of the lack of, in most cases, sufficient testing. Terwee et al. [82] raised in their discussion of the quality of systematic reviews of health related outcome measurement the need for reviewers to make strong recommendations. Our review consists of a large number and wide range of instruments, making it difficult to make those recommendations, particularly as a more thorough evaluation of psychometric properties and quality assessment of included studies was needed. Nevertheless, we have made some suggestions in relation to use of tools depending on their overall quality score. As we chose to include instruments that use surrogate terms and related concepts to women’s childbirth experiences this review presents for researchers and clinicians the diversity of instruments developed. For assessing methodological quality, the COSMIN checklist has newly been developed. It is a detailed and rigorous checklist [75, 76], useful in future systematic literature reviews that have a more narrowed construct of interest, so it could be manageable to do a more in-depth assessment of each instrument comprising both psychometric properties and methodological quality of the development process of each instrument.

## Conclusions

This systematic review provides an overview of existing instruments measuring women’s childbirth experiences and can support researchers to identify an appropriate instrument for their research purpose. Most of the instruments require further validation and reliability testing. Given the plethora of instruments in use in the literature, and the lack of complete testing for many of them, we recommend that researchers do not develop any more new tools, but try to test thoroughly, adapt and improve those that already exist.

Researchers and clinicians need help in finding and selecting the most suitable instrument for their purpose. This makes reviews of measurement instruments important as they aid researchers in finding appropriate, established and tested instruments instead of developing new ones. When different instruments are used to measure the same construct of interest, e.g. women’s experiences of caesarean section, it can become difficult in systematic reviews to compare and statistically report the results. We trust that this review can contribute in helping clinicians and researchers to find the right instrument for their specific context.

## Additional files

Additional file 1: Review protocol. (DOCX 17 kb)

Additional file 2: Search strategy. (DOCX 15 kb)
