# Supplementary material for: Measuring women’s childbirth experiences: a systematic review for identification and analysis of validated instruments
Source: BMC Pregnancy Childbirth. 2017 Jun 29;17:203. doi: 10.1186/s12884-017-1356-y (PMC5492707; doi:10.1186/s12884-017-1356-y)
Supplement: Supplementary file 1 — Review protocol. (DOCX 17 kb) [file 12884_2017_1356_MOESM1_ESM.docx]

# Additional file 1

# Review Protocol for a systematic literature review on validated instruments measuring women’s childbirth experience

Title of the protocol: Identification of validated instruments measuring women’s childbirth experience: a systematic review.

Women’s overall childbirth experience has shown to be an important outcome measure of childbirth. It can have immediate as well as long-term negative or positive effect on women’s life, well-being and health. Therefore, it is vital to identify how women perceive their birth experiences, what contributes to this experience and its continued effect on women’s health, to be able to assure the quality of health services. Risk factors and their importance to the individual woman’s childbirth experience can be measured and the results may/can enable healthcare professionals to improve health care of mothers and their newborns. To achieve this, there is a need for validated questionnaires which can access structural issues and generalize findings.

Aim: To identify and assess validated instruments developed to measure women’s childbirth experience.

#### Review question(s)

We believe that there are a wide range of validated instruments used to measure women’s childbirth experience.

- How many and which are these instruments?
- Which aspects of the childbirth experience do the instruments focus on?
- For what purpose was the instrument developed?
- How has the instrument been validated?
- How has the reliability of the instrument been tested?
- In what country is the instrument developed?
- In what country is the instrument validated?

#### Searches

We will search the following electronic bibliographic databases to identify all relevant studies addressing the research question: PubMed, CINAHL, Scopus, Cochrane library, and PsycINFO.

The search terms will be adapted for use with individual bibliographic databases in combination with database-specific filters, where these are available. Reference lists of identified studies and reviews will be hand searched to identify further relevant papers. Authors will be contacted to further clarify study design and results, if appropriate.

#### URL to search strategy

#### Condition or domain being studied

Validated instruments measuring women’s childbirth experiences.

#### Participants/Population

Inclusion: Women who have gone thorough childbirth. No restriction in age, parity, care setting or country.

#### Intervention(s), exposure(s)

Inclusion: Studies that describe the development and/or validation of an instrument measuring women’s childbirth experience. To be included in the review, an instrument is defined as any structured and validated questionnaire. An instrument may focus on different aspects of the childbirth experience, for example childbirth satisfaction or fear of childbirth. It can also have a more holistic perspective including for example; sense of control, pain, fear, satisfaction with health care professionals etcetera.

Exclusions: Instruments that are not validated. Instruments aimed at partners’ or healthcare professionals’ childbirth experiences.

#### Comparator(s)/control

Not applicable.

#### Types of study to be included

There will be no restriction in study type. The review will include studies which describe validation and/or development of instruments aimed at measuring women’s childbirth experiences. Studies written in English or French will be included.

#### Context

No restrictions will be made due to settings.

#### Outcome(s)

Primary outcome

Identification of validated instrument developed to measure women’s childbirth experience.

#### Data extraction, (selection and coding)

After the initial search, the titles and authorship of all articles will be placed in a database to eliminate the duplicates. The first screening of titles and abstracts will be conducted by one researcher (HN) to exclude all references obtained by searches that are definitely not relevant. Full texts of remaining references will be obtained and screened by two researchers working independently to determine which articles meet the inclusion criteria. Disagreements will be resolved by discussions with a third author.

A data extraction form will be developed for this study and piloted on a small number of papers. We will abstract data concerning the following areas:

1. The name of the instrument.
2. The concept/concepts being measured.
3. Authors, title, year, geographical region.
4. Study design.
5. A description of the instrument regarding domains, items, scoring methods, administration and methods.
6. Participants included in the development and validation of the instrument.
7. The development methods and process.
8. The validation method and process including assessment of face validity, content validity, discriminant validity, and responsiveness.
9. The reliability assessment.
10. Additional relevant information.

#### Risk of bias (quality) assessment

Data extraction will focus on the quality of development and validation of instruments identified, using Terwee et al’s criteria. The quality appraisal of the studies will not be assessed. In the case of discrepancy in opinion, a consensus will be reached by discussion with a third reviewer.

#### Strategy for data synthesis

The results will be presented and summarized for each identified instrument. Analysis and description of the instruments will focus on the purpose of the instrument, the content, development, and validation. We will critical analyze and compare the instruments.

#### Analysis of subgroups or subsets

None planned

#### Dissemination plans

The results will be written up and submitted for publication in a suitable peer-reviewed journal.

#### Contact details for further information

#### Organizational affiliation of the review

University of Gothenburg

#### Review team

Helena Nilvér

Marie Berg

Cecily Begley

#### Collaborators

#### Anticipated or actual start date

18 January 2016

#### Anticipated completion date

3 June 2016

#### Funding sources/sponsors

#### Conflicts of interest

None known

#### Language

English

#### Country

Sweden

#### Subject index terms status

#### Subject index terms

#### Stage of review

#### Date of publication of this revision

#### DOI

#### Stage of review at time of this submission Started Completed

Preliminary searches Yes No

Piloting of the study selection process No No

Formal screening of search results against eligibility criteria No No

Data extraction No No

Risk of bias (quality) assessment No No

Data analysis No No
