# Supplementary material for: Measuring women’s childbirth experiences: a systematic review for identification and analysis of validated instruments
Source: BMC Pregnancy Childbirth. 2017 Jun 29;17:203. doi: 10.1186/s12884-017-1356-y (PMC5492707; doi:10.1186/s12884-017-1356-y)
Supplement: Supplementary file 2 — Search strategy. (DOCX 15 kb) [file 12884_2017_1356_MOESM2_ESM.docx]

# Additional file 2

# Search strategy

### Search string

Instrument* 2. Questionnair* 3. Scale* 4. Tool* 5. Index 6. (1 OR 2 OR 3 OR 4 OR 5) 7. Valid* 8. Develop* 9. Reliab* 10. (7 OR 8 OR 9) 11. Childbirth 12. Birth 13. Parturition 14. Labor or labour 15. Delivery 16. Obstetric* 17. (14 AND 16) 18. (15 AND 16) 19. (11 OR 12 OR 13 OR 17 OR 18) 20. Experience* 21. Satisfaction 22. Fear 23. Perception 24. (19 OR 20 OR 21 OR 22) 25. (5 AND 9 AND 18 AND 23)

### Search strategy for the individual databases

#### PubMed:

Search **((((((((Instrument*) OR questionnaire*) OR scale*) OR tool*) OR index)) AND (((valid*) OR develop*) OR reliab*)) AND (((((childbirth) OR birth) OR parturition) OR (((labor OR labour)) AND obstetric*)) OR ((delivery) AND obstetric*))) AND ((((experience*) OR satisfaction) OR fear) OR perception)**

#### Scopus:

( TITLE-ABS-KEY ( ( ( ( ( ( ( instrument* )  OR  questionnaire* )  OR  scale* )  OR  tool* )  OR  INDEX ) ) )  AND  TITLE-ABS-KEY ( ( ( ( valid* )  OR  develop* )  OR  reliab* ) )  AND  TITLE-ABS-KEY ( ( ( ( ( ( childbirth )  OR  birth )  OR  parturition )  OR  ( ( ( labor  OR  labour ) )  AND  obstetric* ) )  OR  ( ( delivery )  AND  obstetric* ) ) )  AND  TITLE-ABS-KEY ( ( ( ( ( experience* )  OR  satisfaction )  OR  fear )  OR  perception ) ) )

#### CINAHL:

( ( ( ( ( ( ( ( instrument* ) OR questionnaire* ) OR scale* ) OR tool* ) OR INDEX ) ) ) ) AND ( ( ( ( ( valid* ) OR develop* ) OR reliab* ) ) ) AND ( ( ( ( ( ( ( childbirth ) OR birth ) OR parturition ) OR ( ( ( labor OR labour ) ) AND obstetric* ) ) OR ( ( delivery ) AND obstetric* ) ) ) ) AND ( ( ( ( ( ( experience* ) OR satisfaction ) OR fear ) OR perception ) ) ) )

#### PsycINFO:

TI,AB,SU((Instrument* OR questionnaire* OR scale* OR tool* OR index) AND (valid* OR develop* OR reliab*) AND (childbirth OR birth OR parturition OR ((labor OR labour) AND obstetric*) OR (delivery AND obstetric*)) AND (experience* OR satisfaction OR fear OR perception))

#### Cochrane library:

**((((((((Instrument*) OR questionnaire*) OR scale*) OR tool*) OR index)) AND (((valid*) OR develop*) OR reliab*)) AND (((((childbirth) OR birth) OR parturition) OR (((labor OR labour)) AND obstetric*)) OR ((delivery) AND obstetric*))) AND ((((experience*) OR satisfaction) OR fear) OR perception) in Title, Abstract, Keywords (Word variations have been searched)**
